# Development and Laboratory Evaluation of an Online Controlling Algorithm for Precision Tillage

**DOI:** 10.3390/s21165603

**Published:** 2021-08-20

**Authors:** Yashar Sabouri, Yousef Abbaspour-Gilandeh, Aliakbar Solhjou, Mohammad Shaker, Mariusz Szymanek, Maciej Sprawka

**Affiliations:** 1 Department of Biosystems Engineering, Faculty of Agriculture and Natural Resources, University of Mohaghegh Ardabili, Ardabil 56199-11367, Iran; ysabouri@gmail.com; 2 Fars Agricultural and Natural Resources Research and Education Center, Agricultural Engineering Research Department, Agricultural Research, Education, and Extension Organization (AREEO), Shiraz 71555-617, Iran; a.solhjou@areeo.ac.ir (A.S.); m.shaker@areeo.ac.ir (M.S.); 3 Department of Agricultural, Forest and Transport Machinery, University of Life Sciences in Lublin, Street Głęboka 28, 20-612 Lublin, Poland; maciej.sprawka@up.lublin.pl

**Keywords:** compression sensor, pressure limit, depth priority, position sensor, electrohydraulic system

## Abstract

Soil compaction management relies on costly annual deep tillage. Variable-depth tillage or site-specific tillage modifies the physical properties of the soil at the required zones for the growth of crops. In this study, a depth control system was designed for the subsoiler of the tillage at various depths. For this purpose, an algorithm was written to investigate the subsoiler location and soil compaction. A program was also developed to implement this algorithm using Kinco Builder Software to control the subsoiler depth, which was evaluated on the experimental platform. In this study, four compression sensors were used at a distance of 10 cm up to a depth of 40 cm on the blade mounted at the front of the tractor. The data of these sensors were used as the input and compared with the pressure baseline limit (2.07 MPa), and with the priority to select the greater depth, the depth of subsoiler was determined. At all three modes of sensor activation (single, collective, and combined), this system was able to operate the hydraulic system of the tractor and place the subsoiler at the desired depth through the use of the position sensors.

## 1. Introduction

The physical features of the soil can dramatically influence crop yield. The primary responsibility of the soil in plant growth is the mechanical maintenance of the plant while supplying nutrition, water, heat, and air. These tasks are dependent on the soil structure [1]. Soil compaction can affect these tasks and have a negative impact on the crop. The traffic of agricultural machinery, constant-depth tillage, and soil moisture content are among the main factors leading to soil compaction on farms. Trafficking, especially at a soil moisture range of 14–17%, can enhance the apparent specific mass of the soil [2,3]. To eliminate this hardpan layer, deep tillage is often used. The application of this method to all soils (which may be very deep or very shallow) may result in energy loss. Variable-depth tillage can eliminate the hardpan layer via the use of tillage at the required depth. This task requires information regarding the resistance condition of the soil at each depth, the soil’s physical properties, the depth of the hardpan layer, and, hence, the precise depth of the tillage [4,5]. Soil compaction management is often achieved by costly annual tillage. Variable-depth tillage or site-specific tillage only refine the physical properties of the soil at places where the tillage is required for plant growth. This approach can reduce costs and the consumption of labor, fuel, and energy. Moreover, there is very little to gain from tilling deeper than the compacted layer [6].

Some experiments have been conducted in three different soil textures to compare the energy required for site-specific tillage with that of constant-depth tillage. The tillage depth to break the hardpan layer was determined based on a cone index higher than 2.07 MPa. The results indicated 50% energy saving and 30% lower fuel consumption upon applying site-specific tillage in loamy sand soil [7]. The position and thickness of the hardpan layer should be determined for optimal tillage. Moreover, tillage at depths below the compact layer has few advantages and can be harmful in clay and loam soils [6]. The tools used to detect the hardpan layer can be classified into horizontal and vertical. These tools use a force transducer to measure the diffusion resistance of the soil in combination with the soil profile. The depth at which the force required to penetrate is higher than the root growth limit is called the hardpan layer depth. The difference between these two types of tools is in their method of passing through the soil profile [8]. Numerous farmers conduct tillage at a constant depth and at the maximum depth of the tools. However, studies have shown that the depth and resistance of the hardpan layer differs between farms or between regions of the same farm [9,10]. Adamchuk et al. [11] presented an algorithm to determine the presence of hardpan layers in a farm. They compared the linear distribution of the pressure (pressure on the blade tip estimated by two series of strain sensors) with the real force on the blade tip and concluded that the horizontal component of the force has higher sensitivity in detecting the hardpan layer. Numerous researchers, such as Gorucu et al. [12], Mouazen et al. [13], Chung et al. [14], and Furriel et al. [15], also reported continuous measurement of the soil mechanical resistance. Abbaspour-Gilandeh [16] constructed and evaluated a mobile device to measure the mechanical properties of the soil. The device had four arms and collected the data at four depths (10, 20, 30, and 40 cm). The soil resistance was measured using an octagonal transducer. Initial farm experiments indicated the success of this system in measuring the mechanical resistance of the soil.

Gorucu et al. [17] conducted experiments on variable-depth tillage based on the soil compaction reference data for coastal plains. The variable-depth tillage was compared with no tillage and conventional tillage, and the relationships between the tillage depth, soil EC, crop response, and cotton yield were investigated. The results indicated that the required depth of tillage is lower than the conventional tillage depths. Variable-depth tillage led to 56.4 and 33.8% savings in energy and fuel consumption, respectively.

Khalilian et al. [18] applied the technology of variable-depth tillage in coastal plains. A subsoiler stem was equipped with GPS to control its depth during motion, and the test was carried out according to the physical parameters of the soil. An electrohydraulic operator and control valves were used to control the relative direction and move the four-row subsoiler wheels up and down. The tests were conducted in two years to compare the effects of the variable-depth tillage, constant-depth tillage, and no tillage on the soil parameters and crop yield. Based on the data, about 75% of the tested region required tillage at depths less than the recommended depth. Declines of 42.8 and 28.4% were observed in the energy and fuel consumption, respectively, when using variable-depth tillage.

Several studies have been undertaken on the continuous measurement of soil strength at multiple depths. In addition, map-based equipment for changing tillage depth during operation was developed by Clemson researchers. However, currently, there is no equipment available to automatically control the tillage depth to match the soil’s physical properties [19].

Methods for controlling the depth of tillage implements can be divided into two categories: sensor based and map based. Most research falls into the map-based category. In this method, in the first stage, soil compaction and possibly other related characteristics are measured and the soil compaction map is prepared according to this information and communication with GPS. Then, in the second stage, according to the obtained map, the site-specific subsoiling operation is performed. In the sensor-based method, both of the above steps are summarized in one step, and collection of data relating to soil compaction and subsoiler control are undertaken simultaneously. This research is based on the second strategy and uses sensors installed at the front of a tractor to collect soil compaction data and, at the same time, control the depth of the subsoiler mounted on the back of the tractor and respond to the control system to effectively remove the hardpan. 

Due to the lack of development in precision tillage, further research is required to investigate and compare the advantages and disadvantages of these two systems under different agricultural soil conditions, in addition to the systems’ construction costs, energy requirements, and the amount of compaction resulting from their use. Thus, the aims of the current research were to:

1. Develop an algorithm to detect the existence of the hardpan layer and its depth using data from compaction sensors mounted on the instrumented blade that is installed on the front of tractor;
2. Develop a program for the position of the subsoiler, mounted on the tractor, in a closed-loop controlling circuit;
3. Design and construct the hydraulic and mechanical parts of the depth-control system;
4. Investigate the placement accuracy of the subsoiler at the intended depth with respect to compaction and position sensors’ data.

## 2. Materials and Methods

### 2.1. Field and Soil Properties

This project was carried out in the Aliabad education center at a distance of 10 km from Saadatshahr, in the North of Fars province, Iran. In this research, a tractor (John Deere 3140, 97 hp, hydraulic sys., 190 bar, 47 L/min, Moline, IL, USA) was employed.

Samples of field soil were taken to determine soil texture and measure soil moisture at four different depths (5–15, 15–25, 25–35, and 35–45 cm) with three replications. Samples were taken from the center of each depth and placed in an oven at 105 °C for 24 h to measure soil moisture content.

### 2.2. Algorithm Development

An algorithm was developed to detect the depth of the hardpan layer. To determine the position of the hardpan layer inside the soil, the compaction limit value was considered to be equal to 2.07 MPa [12]. 

**Figure 1:**
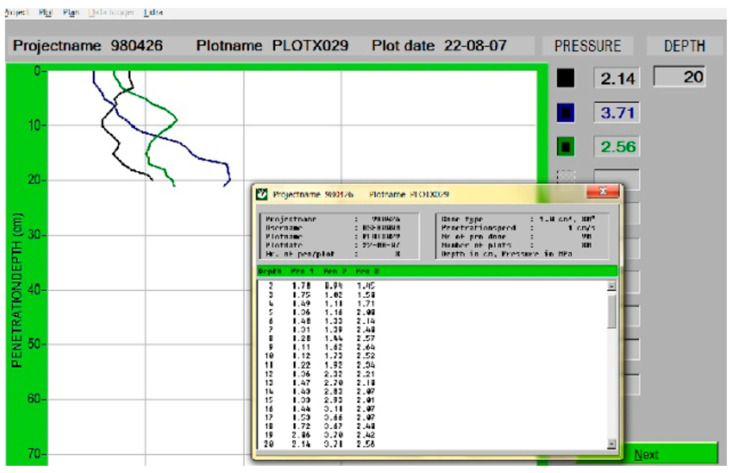
An example of soil cone penetrometer data (MPa) at depths up to 20 cm.

First, 90 penetrations were performed experimentally (up to a depth of 20 cm) in three different places (5 × 20 m width and length) on the field using a vertical soil cone penetrometer (Eijkelkamp penetrologger, Giesbeek, The Netherlands) equipped with a cone with a vertex angle of 30 degrees and a cross section of 130 mm^2^. The penetration rate was 1 cm/s (Figure 1).

Then, each datapoint was examined separately on a computer as follows:

1. First, all of the data related to the soil penetration were arranged in a table with the two characteristics of depth and compaction value.
2. According to the limit value (2.07 MPa) that was considered for compaction, values above this limit were considered to be a hard layer and separated.
3. The depth corresponding to each of these specified values was determined.
4. In each penetration, the maximum depth was determined based on the compaction values higher than the limit value.

In this algorithm, the priority of selecting a greater compression depth was considered for compression values higher than the limit value; that is, if at a specific soil depth, the compaction values exceeded 2.07 MPa at more than one depth, the priority was to select the greater depth. In some penetration measurements, the compaction values increased without increasing the depth, which may be due to the presence of obstacles such as rocks in the soil, which were omitted.

### 2.3. Programing and Algorithm Improvement

A program was developed in this research to control the subsoiler depth and evaluate it under laboratory conditions. The program should be able to determine the soil compaction based on the data provided by the sensor, and compare them with a limit value to make the best decision on the depth of the subsoiler mounted on the back of the tractor.

The program was written using Kinco Builder Software according to the mentioned algorithm and was installed on a K205 PLC under the Modbus Protocol. By applying manual values rather than compression sensors, the system’s response to these compression values was confirmed. Considering that this system was able to respond appropriately to the compression stimulus, another 40 datapoints were collected by the soil cone penetrometer up to a depth of 40 cm and, according to the mentioned procedure, the existing algorithm and the written program were developed.

**Figure 2:**
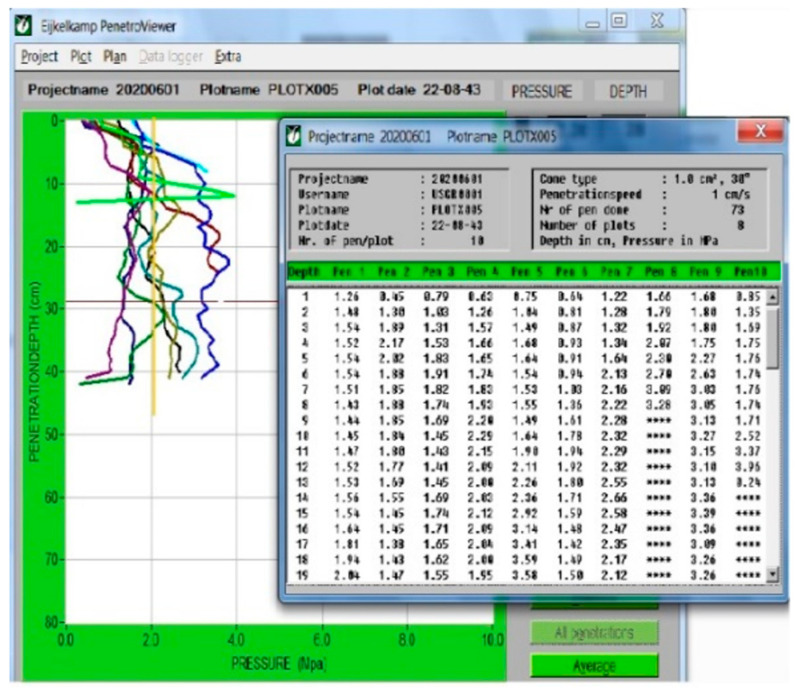
An example of soil cone penetrometer data at different depths along with the baseline (2.07 MPa).

Figure 2 shows an example of data related to soil compaction at different depths (graphically and numerically) along with the baseline (2.07 MPa).

**Figure 3:**
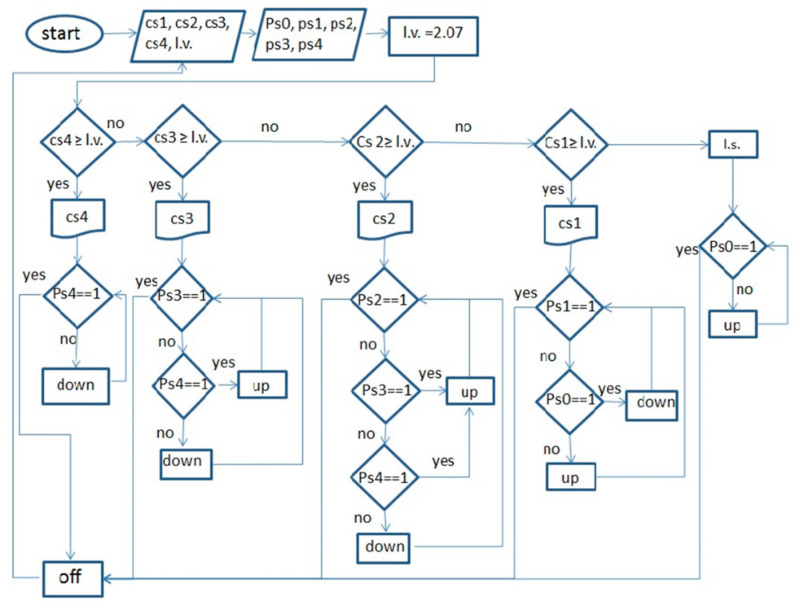
Flowchart for determining the hardpan layer, selecting the appropriate depth, activating the solenoid valve, and confirming the hydraulic jack position based on sensor data (cs1: compaction sensor at 10 cm depth, …, l.v.: pressure limit value (2.07 MPa), Ps0: zero position sensor on the rail (transport position), Ps1: position sensor that indicates the subsoiler depth in 10 cm, …, “up and down” are the settings of the solenoid valve that indicate the direction of the jack movement).

In this research, four sensors were used at specific depths (10, 20, 30, and 40 cm); thus, the algorithm was defined and implemented, as shown in the flowchart in Figure 3.

### 2.4. The System Used to Measure the Mechanical Resistance of the Soil

**Figure 4:**
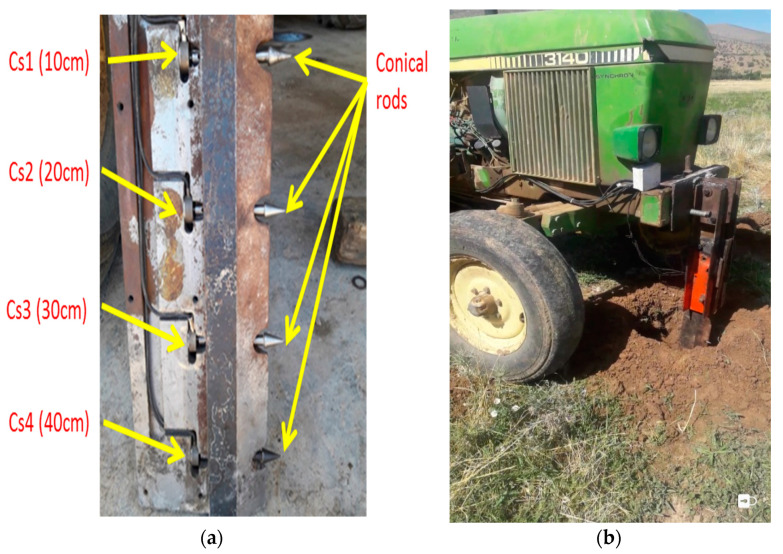
(**a**) Loadcells and conic bars of pressure mounted on the blade, (**b**) the blade’s position at the front of the tractor.

In this research, a blade with four sensors that was constructed in the Biosystem Department of University of Mohaghegh Ardabili was used with minor modifications [8]. The instrumented blade descends to a depth of 40 cm in the soil. The soil force is applied by conical rods to the loadcells. In pretests conducted to investigate the potential problems of using an instrumented blade in this field, the shear pin was broken in some cases. It was decided to change the position of the shear pin on the blade, and the conical rod length (the distance between the shear pin and the pivot pin) was changed from 60 to 350 mm, and the length of the conical rod was changed from 102 to 80 mm. A support base was made and installed at the front of the tractor to install the equipped blade (Figure 4).

### 2.5. Design and Fabrication of the Hydraulic System

**Figure 5:**
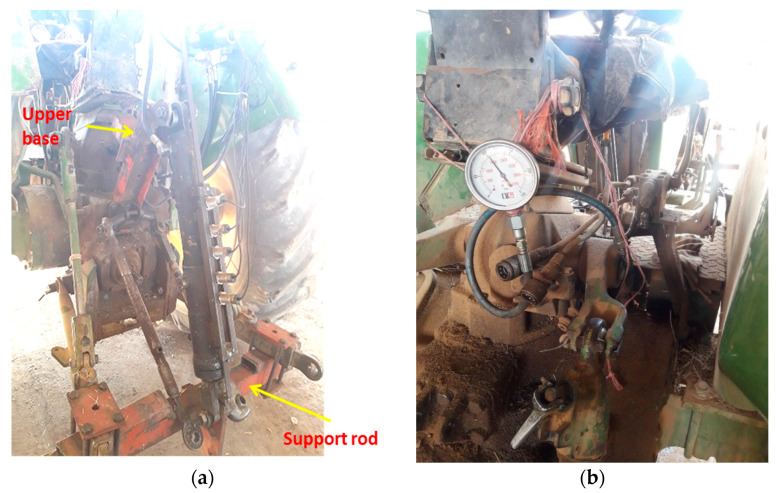
(**a**) Hydraulic jack with its junctions, (**b**) measurement of the pressure at the valve inlet of the tractor position.

The height of the tractor arms was controlled by a hydraulic jack which is attached to the tractor by a separate base at one end and to a support rod mounted on the lower arms of the tractor at the other end (Figure 5a).

The jack attachment position overlapped the middle arm of the tractor. To solve this problem, a pair of middle arms were used on both sides of the hydraulic jack. In this manner, space was provided between the arms to place the hydraulic jack.

The cross-sectional area of the hydraulic jack was calculated by considering the contact surface of the pair of selected C-shaped subsoiler tines with the soil, levering on the location of the hydraulic jack and the pressure required by the tractor hydraulic system (90 bar). Calculations related to the critical load of elastic buckling were performed using the Euler equation considering the location of the hydraulic jack, its joints, a safety factor of 3.5, and its course (450 mm). A hydraulic jack of 65/35 was selected for this system.

The outputs (A, B) of the solenoid hydraulic valve used in this circuit (Rextour: 4WE 10 G-33/CG12N9Z4) were connected to two ports of the two-way depth control hydraulic jack. Part T of the valve was also connected to the tractor hydraulic tank (gearbox). The inlet hose to the position and traction control valve of the tractor was separated and installed directly to the inlet port (p) on the valve. The pressure and flow rate at this point were 105 bar and 30 L/min at 2500 rpm, respectively (Figure 5b).

### 2.6. Design and Fabrication of the Hydraulic Jack Control Rail

**Figure 6:**
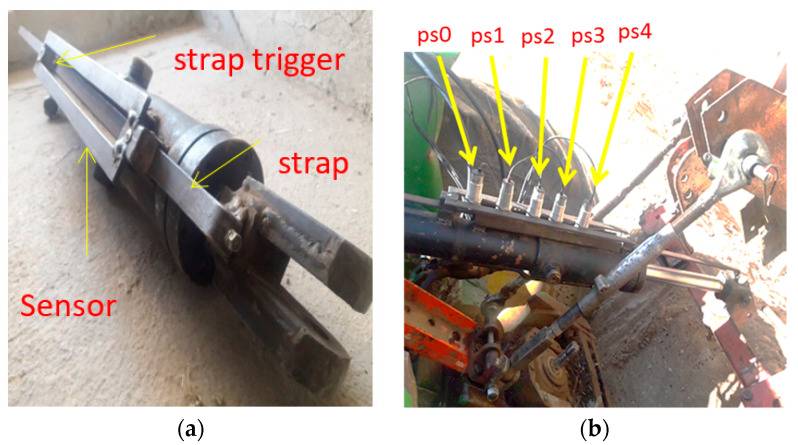
(**a**) Hydraulic jack with rails related to position sensors and metal belt (strap) with sensor-actuator trigger, (**b**) hydraulic jacks and position sensors on the rails on the tractor.

To install the position sensors and detect the movement of the hydraulic jack arm, a rail with a span distance of 30 mm and dimensions of 500 × 70 mm was made and installed on one side of the hydraulic jack, parallel to the piston movement axis. The latch strap was attached inside this rail and to the end of the piston handle (Figure 6a). A cylindrical trigger, with a diameter of 7 mm and a height of 5 mm, triggered the sensors, and was placed at the appropriate point on the strap. Five magnetic position sensors (IPS-210-CD-30, DC/NC (Normal Close), input of 10–30 VDC, made by Tabriz Pajooh Company, Tabriz, Iran) were installed on the rail as position feedback sensors in the control circuit for precise control of the subsoiler position.

The distance between the position sensors on the rail was approximately 75 mm per 100 mm of subsoiler (hydraulic jack arm) displacement (Figure 6b). The toggle must be at a maximum distance of 10 mm from the sensor head and at a distance of 10 mm from the center of the sensor (±10 mm) along the motion direction.

### 2.7. Control Panel

**Figure 7:**
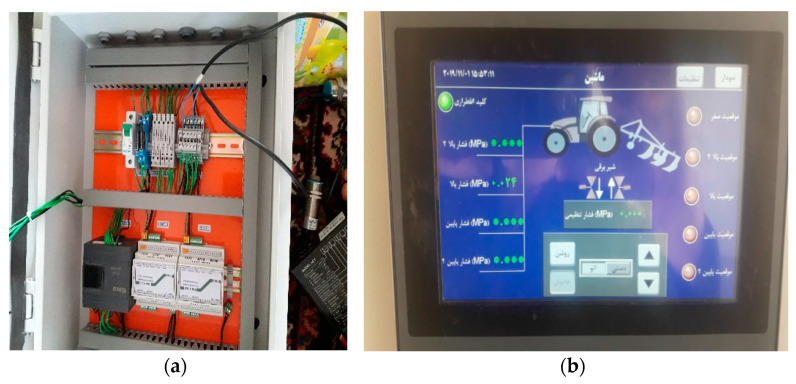
(**a**) Internal parts of the control panel and its related wiring, (**b**) HMI display and its active items.

The control panel of this system included the transmitter, controller, protective fuses, touch screen, and emergency shutdown key. In the unit used for measurement and comparison, a logic controller (PLC, Kinco Model: k205-16DR/Source 24VDC 5W MAX, Shenzhen, China) was used with a maximum input speed of 50 kHz in the program. Using two transmitters (Weighting indicator & Transmitter: TM 1022, 24 V DC, RS 485, Shanghai, China), the values measured by the loadcells were converted into a standard signal (4–20 mA) and sent to the controller. The power required for this circuit was 24 V. The map of the wiring and connections of the circuit components, which represented the interface between the soil compaction sensors, the position sensors on the cylinder, and the solenoid hydraulic valve with the components inside the control panel, was designed in AutoCAD software, executed on the panel, and then installed on the tractor (Figure 7a).

Upon facing an obstacle or defect in the hydraulic system, the emergency mode of the system was able to be activated by the driver, and the subsoiler returned to the zero point of movement (highest point). The output (USB) of the control panel was used to store information received from compaction sensors and subsoiler position sensors. The touch-panel display was designed in three columns, which were related to the position of the subsoiler (in five moving positions), a column for setting the pressure limit value and determining the manual or automatic modes, and a third column related to the emergency key light and the compression pressure received from the corresponding sensors (Figure 7b).

### 2.8. Evaluation of the Control System

After installing the control panel, hydraulic valve and jack, compression sensors, and position control sensors, the subsoiler was connected to the tractor arms and transported to the test platform for evaluation.

**Figure 8:**
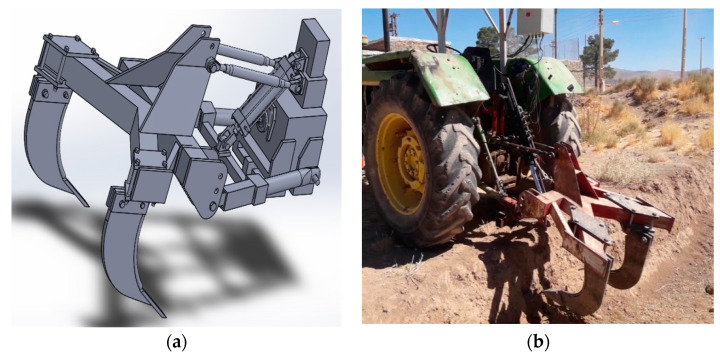
Subsoiler and the hydraulic system mounted on the tractor: (**a**) designed form, (**b**) real form at work at a depth of 40 cm on the platform.

The platform was measured and marked at a distance of 10 cm from the soil surface. The control panel was activated, and the pressure limit value was manually entered. The controller reaction, solenoid valve, and, finally, the position of the subsoiler, were evaluated by selecting the automatic mode and applying pressure in three different modes (single, combinational, and both) with the help of tools on the conical rods of the compression sensors related to different depths. The evaluation of the control system response was undertaken using three modes:

(1) System response to single and separate excitation of the compression sensor: In this section, the compaction sensors on the instrumented blade were excited separately and sequentially. Following the investigation of the reaction of the control system to the related sensor excitation, and replacement of the subsoiler to the zero position, the test was conducted for the next sensor.
(2) Combinational excitation of compression sensors (successive with and without interference): In this section of the test, compaction sensors were excited sequentially and continuously, and sequentially. In some cases, exciting of the next compaction sensor was undertaken before the replacement of the subsoiler at the zero position (i.e., with interference between the end of the previous sensor action and beginning of the next sensor action).
(3) Simultaneous excitation of compression sensors: In this section, all compaction sensors were excited at approximately the same time to study the prioritization and accuracy of the control system when selecting the appropriate compaction sensor data and placing the subsoiler at the related depth (Figure 8).

In these tests, the hydraulic jack was moved to the desired depth and, when the depth corresponding to the excited compression sensor was reached, the tab on the strap attached to the jack arm stimulated the corresponding position sensor on the hydraulic jack and sent a message to the control system. This inactivated the solenoid in the direction of movement and stopped the hydraulic jack in the same position. By removing the pressure on the conical rod, the control system, which does not receive pressure greater than the limit value from any of the soil compaction sensors, activates the corresponding valve solenoid until it receives a signal from the zero position sensor (transport position).

## 3. Results

Data obtained from the soil compaction sensors and positioning sensors of the hydraulic jack were investigated to evaluate the performance of the depth control system in three modes on a test platform.

### 3.1. Results Related to the First Mode (Separate and Single Excitation of Compression Sensors)

**Figure 9:**
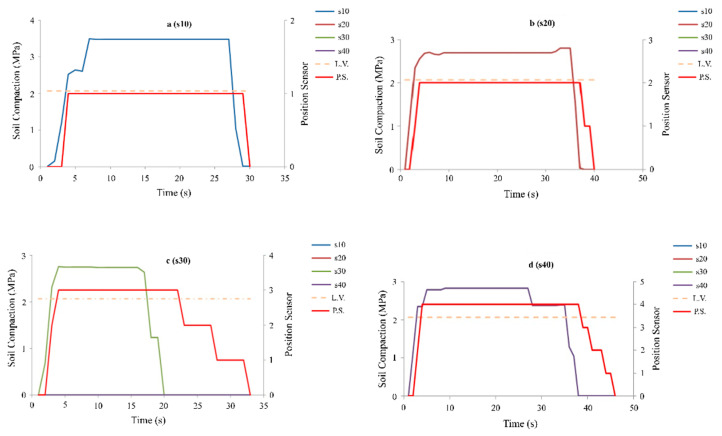
Operating mode of the system and feedback (red line) in the first mode, and the excitation of the compression sensors.

Figure 9 shows the operating mode of the system in the first mode and the excitation of the compression sensors. By exceeding the pressure sensor pressure line from the pressure limit value (2.07 MPa, dashed line), the controller activated the hydraulic valve solenoid to operate the hydraulic jack. When the actuator reached the position corresponding to the sensor, the valve was immediately disconnected, and the jack (subsoiler) was stopped in the desired position. By reducing the pressure of the compression sensor below the limit value, the hydraulic jack was closed and stopped at the zero position (transport position).

### 3.2. The Second Case Combinational Excitation of Several Compression Sensors

**Figure 10:**
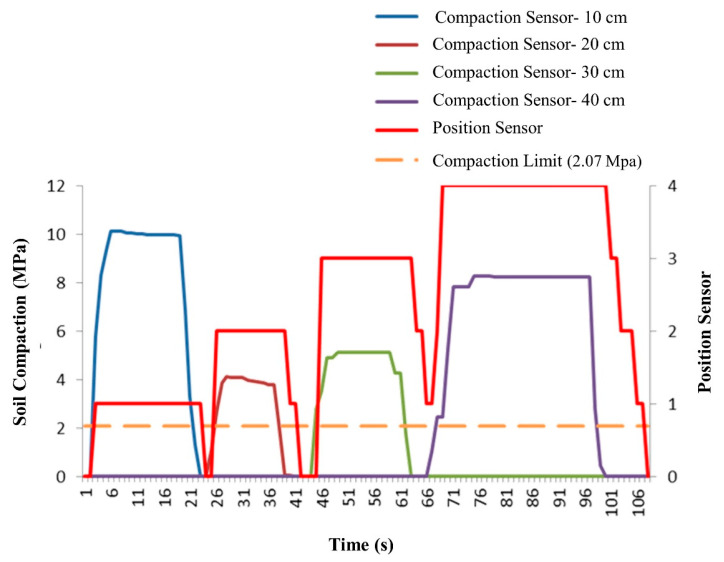
Combinational performance of compression sensors (depths of 10, 20, 30, and 40 cm), limit density (2.07 MPa), and the position of the subsoiler position sensor.

In this stage of the experiment, the reaction of the control system was investigated via the excitation of more than one compression sensor with different values of compression pressure. Figure 10 shows the excitation of compression sensors from the first depth (10 cm) to a depth of 40 cm. Relevant position sensors indicated the location of the subsoiler at appropriate depths. By disconnecting the pressure on one sensor and exciting the following sensor, and reaching a pressure above the baseline value, the sensor shows the position of the subsoiler at a new depth. In the first two cases, the subsoiler ultimately returned to the zero position (transport). When returning from the third position and before the subsoiler reached the zero position, the pressure value of the fourth sensor exceeded the baseline level, and the subsoiler was immediately placed in the fourth position. 

### 3.3. The Third Case; the Simultaneous Excitation of Compression Sensors

In this case, the sensors were simultaneously activated. The difference between this case and the previous two cases is that, in addition to separately examining the values received from the compression sensors and comparing them with the baseline, the second performance metric of the system, namely, the sensor prioritization, was also examined. Table 1 shows the values received from the quadruple compression sensors and the feedback reaction of the five position sensors. 

**Figure 11:**
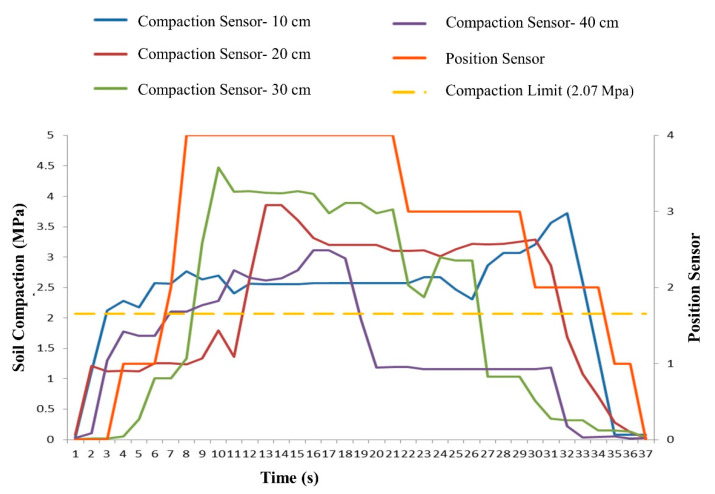
Combinational performance of compression sensors (depths of 10, 20, 30, and 40 cm), limit density (2.07 MPa), and the location of the subsoiler position sensor.

As can be seen in Table 2, the density of the first sensor exceeded the baseline pressure (2.07 MPa) at t = 3 s of the test. The controller stabilized the subsoiler position in position one at t = 4 s (within 1 s). At t = 7 s, the density of the fourth sensor increased; thus, at t = 8 s, the fourth position was stabilized by the controller, and the subsoiler was placed at a depth of 40 cm. Note that, at this time, compression sensors one and four both had values above the baseline. At t = 9 s, the third compression sensor, and at t = 12 s, the second compression sensor, exceeded the baseline limit. It should be mentioned that, in this situation, although all four sensors exceeded the baseline limit and the compression values for the second and third sensors were higher than that for the fourth sensor, according to the prioritization program, the system maintained position four for up to 21 s. The sensor density lowered below the baseline limit at t = 19 s (this time difference is due to the toggle movement between the two position sensors). At this time, the value of the third sensor was above the baseline. At t = 27 s, the third compression sensor fell below the baseline. At t = 30 s, the second position sensor was stabilized. At t = 34 s, all compression sensors fell below the baseline. Figure 11 shows the working modes of these sensors. 

## 4. Discussion

Based on the results related to the first mode (separate and single excitation of compression sensors), it can be seen that the time when the hydraulic jack moved to the desired position (the total time delay of the control system, valve, and hydraulic jack) was less than the time the jack was in the zero position (return position). Due to the same time delay of the control valve and the hydraulic valve during the go and return modes, the effective factor in this difference is the weight of the subsoiler and the internal leakage of the tractor hydraulic system at high pressures. The weight of the subsoiler helped when lowering, while having a negative impact when returning, thus delaying the placement of the system.

The second case, of combinational excitation of several compression sensors, indicates that the control system is continuously monitoring the circuit. Upon seeing a value above the baseline in another sensor, it immediately changed the movement direction of the hydraulic jack and corrected the position of the jack. 

Another point is the stepwise movement of the hydraulic jack during the return and closing of the hydraulic jack, which is not due to a stoppage, but is rather due to the number of position sensors, and the impossibility of detecting all of the toggle paths by the sensors. Thus, the control system determined the instantaneous position based on the last excited sensor; that is, from the start of the actuator toggle from the front of the current position sensor (last excited sensor) to the new sensor and its excitation, the current sensor is the basis for determining the position of the subsoiler.

During the simultaneous excitation of the compression sensors, which is also the most important part of the control system performance test, this control system exhibited acceptable results despite the low return speed of the hydraulic system (travel time from one sensor to another sensor during the return), which is due to the operation of the pump and the hydraulic circuit of the tractor. It achieved both objectives of this design: compression detection and placement of the subsoiler at the desired depth.

In this system, the working temperature was not measured; however, during the work, the increase in temperature was clearly visible. Considering that this tractor was equipped with an oil radiator, it did not cause any functional problems for the system. The pressure measured during the test (at the outlet of the tractor hydraulic system and the inlet of the control system) decreased slightly, possibly due to the increase in operating temperature (the hydraulic system oil was changed at the beginning of the test) and the depreciation of the tractor hydraulic pump (due to obsolescence). 

Most previous studies were undertaken to perform precision tillage operations in terms of fuel consumption, required power, crop yield, and plant root expansion in the vertical direction. Limited research has been conducted on the depth control of precision tillage implements [19,20,21]. All of these studies use a separate data collection system for soil strength and soil compaction measurement. A comprehensive system that can simultaneously measure soil strength, in addition to processing the data online and changing the depth of a moving tillage implement, was not previously developed.

This research was conducted to investigate the possibility of detecting the depth of the hardpan layer in the soil using an algorithm. The obtained results show the efficiency of this method in detecting the hardpan layer, and are consistent with the results of research conducted by researchers such as Gorucu et al. [12]. In addition, previous researchers generalized their algorithm, from the method of using a cone penetrometer in situ, to the method of a moving cone permeation meter.

The current research is one of the few studies conducted in the field of precision tillage for online precise depth control of tillage tools. By receiving the necessary information for precision tillage, such as the depth of the soil hardpan layer at different locations on agricultural land, the online control algorithm designed in this research changes the position of the agricultural equipment relative to the land surface, as needed. The advantage of the device is that it can be installed on a variety of conventional tractors, without a change in the structure or operation of the three-point connection mechanism, or the hydraulic system of the tractor.

## 5. Conclusions

This study aimed to develop and evaluate an online control algorithm for precision tillage. An electro-hydraulic depth control system was mounted on a tractor to change the depth of tillage implements based on the location of the hardpan layer. An algorithm was developed to detect the depth of the hardpan layer. Moreover, a program was developed to implement the algorithm using Kinco Builder Software to control the subsoiler depth. The test results showed the ability to receive data, detect the hardpan, and decide on the placement of the subsoiler at the appropriate depth based on information from compression sensors located on the blade and at different depths. The system was able to react to changes in the compression values of the sensors upon individual or collective sensor excitement, detect the appropriate position, and deploy the subsoiler at the desired point. This device can be easily installed and operated with minimal alterations to the structure of the tractor.

The results of the test platform confirmed that the excitation of the compression sensors resulted in reliable depth control. This system can be recommended for variable-depth tillage of agricultural lands.

## Figures and Tables

**Table 1:** Soil characteristics and the mean of soil moisture content (%) at different depths.

pH	EC	Sand %	Silt %	Clay %	Soil
8.1	0.47	20.4	44.4	35.2	clay/loam
Depth	10 cm	20 cm	30 cm	40 cm	
Mean M.C. (%)	11.37	10.11	11.12	12.07	

**Table 2:** Data received from the quadruple compression sensors and the feedback reaction of the five position sensors.

Time (s)	Compaction Sensor at 10 cm (MPa)	Compaction Sensor at 20 cm (MPa)	Compaction Sensor at 30 cm (MPa)	Compaction Sensor at 40 cm (MPa)	Position Sensor Condition	Compaction Limit (2.07 MPa)
1	0.03736	0.08265	0.01184	0.02548	0	2.07
2	1.11920	1.21444	0.01438	0.10446	0	2.07
3	2.11919	1.12604	0.01440	1.29654	0	2.07
4	2.28216	1.13473	0.05418	1.77510	1	2.07
5	2.17623	1.12685	0.33541	1.70189	1	2.07
6	2.57037	1.25138	1.00351	1.71039	1	2.07
7	2.56744	1.25257	1.00353	2.10356	2	2.07
8	2.76520	1.24103	1.33542	2.10408	4	2.07
9	2.63620	1.33119	3.22920	2.20716	4	2.07
10	2.69812	1.79335	4.46871	2.27932	4	2.07
11	2.40171	1.36334	4.07384	2.78833	4	2.07
12	2.55960	2.65314	4.08625	2.66208	4	2.07
13	2.55861	3.85502	4.05372	2.61228	4	2.07
14	2.55861	3.85502	4.04840	2.65149	4	2.07
15	2.55872	3.60896	4.08069	2.78613	4	2.07
16	2.57392	3.31730	4.03953	3.10784	4	2.07
17	2.56804	3.20370	3.71720	3.11006	4	2.07
18	2.56804	3.20370	3.88834	2.97470	4	2.07
19	2.56804	3.20370	3.88712	1.98250	4	2.07
20	2.57061	3.20328	3.72187	1.18807	4	2.07
21	2.57088	3.10163	3.78382	1.18994	4	2.07
22	2.57056	3.10417	2.53899	1.18994	3	2.07
23	2.66991	3.10862	2.33954	1.15999	3	2.07
24	2.66979	3.01810	2.99905	1.15995	3	2.07
25	2.46993	3.13020	2.94008	1.15993	3	2.07
26	2.30699	3.21541	2.93944	1.15991	3	2.07
27	2.86699	3.21192	1.03864	1.15995	3	2.07
28	3.06993	3.21463	1.03789	1.15982	3	2.07
29	3.06993	3.25542	1.03676	1.15989	3	2.07
30	3.20754	3.28719	0.63644	1.15988	2	2.07
31	3.56180	2.86440	0.34728	1.18807	2	2.07
32	3.72054	1.68936	0.31465	0.22220	2	2.07
33	2.57755	1.08071	0.31955	0.03521	2	2.07
34	1.37174	0.70680	0.14856	0.04477	2	2.07
35	0.07758	0.28186	0.14869	0.05491	1	2.07
36	0.07758	0.12742	0.13470	0.01906	1	2.07
37	0.07758	0.02932	0.03477	0.02542	0	2.07

## Data Availability

The data presented in this study are available on request from the corresponding author.
